# Patterns, mechanism of injury and outcome of pediatric trauma at a level 1 trauma centre: a descriptive retrospective analysis

**DOI:** 10.3389/fped.2023.1084715

**Published:** 2023-04-28

**Authors:** Amani N. Alansari, Ahammed Mekkodathil, Ruben Peralta, Temur Baykuziyev, Nour W. Z. Alhussaini, Mohammad Asim, Ayman El-Menyar

**Affiliations:** ^1^ Department of Pediatric Surgery & Trauma Surgery, Hamad Medical Corporation, Doha, Qatar; ^2^ Clinical Research, Trauma and Vascular Surgery, Hamad Medical Corporation, Doha, Qatar; ^3^ Trauma Surgery Section, Hamad General Hospital (HGH), Doha, Qatar; ^4^ Department of Surgery, Universidad Nacional Pedro Henriquez Urena, Santo Domingo, Dominican Republic; ^5^ Department of Anesthesiology, ICU and Peri-operative Medicine, Hamad Medical Corporation, Doha, Qatar; ^6^ Department of Public Health, Qatar University, Doha, Qatar; ^7^ Clinical Medicine, Weill Cornell Medical College, Doha, Qatar

**Keywords:** epidemiology, pediatric, trauma, injury, mortality

## Abstract

**Background:**

There is a gap in knowledge on the epidemiology of pediatric trauma in the developing countries. We aimed to describe the injury pattern, mechanism of injury (MOI), and outcomes of pediatric trauma in a level 1 trauma centre in one of the Arab Middle Eastern countries.

**Methods:**

A retrospective analysis of pediatric injury data was conducted. All trauma patients (<18 years old) requiring hospitalization between 2012 and 2021 were included. Patients were categorized and compared based on the MOI, age-group and injury severity.

**Results:**

A 3,058 pediatric patients (20% of the total trauma admissions) were included in the study. The incidence rate in 2020 was 86 cases per 100,000 pediatric population in Qatar. The majority were male (78%) and the mean age was 9.3 ± 5.7 years. Nearly 40% had head injuries. The in-hospital mortality rate was 3.8%. The median injury severity score (ISS) (interquartile range; IQR) was 9 (4–14) and Glasgow coma scale (GCS) was 15 (IQR 15–15). Almost 18% required Intensive Care admission. Road Traffic Injuries (RTI) were more frequent in 15–18 years old whereas ≤4 years group was mostly injured by falling objects. The case fatality rate was higher among females (5.0%), and in 15–18 years (4.6%) and <4 years (4.4%) group. Pedestrian injuries were more lethal among the MOI. One fifth had severe injury with a mean age of 11 ± 6 and 9.5% had ISS of ≥25. Predictors of severe injury were age (10 years old and above) and RTI.

**Conclusion:**

Almost one-fifth of the trauma admissions at the level 1 trauma centre in Qatar is due to traumatic injuries among the pediatric population. Developing strategies that are based on understanding the age- and mechanism-specific patterns of traumatic injuries among the pediatric population remains crucial.

## Introduction

Pediatric trauma is associated with significant morbidity and mortality and remains one of the public health concerns worldwide, particularly in the developing countries. Traumatic injuries from all causes result in 8.4%–9.3% mortality in children and appear to be on the rise in the Arab Gulf States, mirroring what is happening in higher income countries such as the USA (1). According to the World Health Organization (WHO), almost 2,000 children under the age of 14 years die every day around the globe, and the disabilities associated with traumatic injuries can have long-lasting impacts on all aspects of victims' lives (2). Some form of disability was reported in nearly half of pediatric trauma patients below the age of 12 years presenting to the emergency department (ED) (3). Road traffic collisions, drowning, burns, falls and poisoning contributes 60% of all pediatric trauma mortality (3). It is essential to consider a child's age when developing injury prevention strategies as growing and developing children have different needs.

The nationally representative trauma registry data in Qatar revealed that the incidence of severe pediatric trauma was 58 per 100,000 pediatric cases presented to the ED 10 years ago (4).

Understanding trauma epidemiology is critical for developing region-specific injury prevention policy. There are few but outdated epidemiological studies on pediatric trauma in Qatar (4–6). Updated information is required to assess the current strategies and to fill the gaps for better management and outcome of trauma within the pediatric population. The aim of the present study is to describe the pattern of injuries, management, and clinical consequences of traumatic injuries in the pediatric population in Qatar, an example of the rapidly developing Arab Middle Eastern countries.

## Materials and Methods

A retrospective analysis of pediatric injury data obtained from the Qatar Trauma Registry (QTR) between 01 January 2012 and 31 January 2021 was conducted. All pediatric patients below the age of 18 years admitted to the Hamad Trauma Center (HTC) were consecutively included in the study. Severely injured patients who died at the scene of injury before arriving at the hospital; and those with mild injuries who were treated and discharged from the Emergency Department (ED) of Hamad General Hospital (HGH) or Primary Health Care Centers (PHCC), were excluded from the study. The pediatric trauma care in Qatar has been split between Sidra medicine center and the HTC since March 2019, however, both are sharing the data with the QTR. The HTC is the only level 1 trauma facility in Qatar, and it provides treatment for moderate to severe injuries for trauma patients at no cost to the patients. The QTR is compliant with both the National Trauma Data Bank (NTDB) and Trauma Quality Improvement Program (TQIP) of the American College of Surgeons Committee on Trauma (ACS-COT). HTC has obtained the Accreditation Canada Distinction Program in the year 2014 (7). In the present study, the definition of traumatic injuries was based on the QTR data which follow the International Classification of Diseases, Ninth Revision (ICD-9) codes and later upgraded to version 10 (ICD-10) codes. Ventilator associated pneumonia (VAP), sepsis, and acute respiratory distress syndrome (ARDS) were defined previously (8).

The data collected includes patient demographics (age, gender, and nationality), type of trauma (blunt or penetrating) and mechanism of injury (MOI) (traffic-related, pedestrian, fall, fall of objects and others). It also recorded the vital signs (systolic and diastolic blood pressure at ED, and respiratory rate), injured body regions (head, chest, abdomen, pelvis, extremities) and injury severity measures [Abbreviated Injury Scale (AIS), Injury Severity Score (ISS), Glasgow Coma Score (GCS)]. In addition, data included interventions like intubation, orthopedic interventions, debridement, craniotomy or craniectomy, exploratory laparotomy, and chest tube insertion. Moreover, blood transfusion, massive transfusion protocol (MTP), in-hospital complications (VAP, sepsis, ARDS), ventilatory days, intensive care unit (ICU) days and hospital length of stay (HLOS) were documented. The all-cause hospital mortality rate, and all case fatality rate (CFR) were presented.

### Statistical analysis

Categorical data were presented as counts and percentages. The *χ*^2^ test was used to compare categorical variables. Fisher's Exact Test was used when the expected count was less than 5 in any of the study groups, for example, extremity injuries, interventions such as internal fixation, craniotomy or craniectomy, exploratory laparotomy, chest tube insertion, MTP, and complications such as VAP, sepsis and ARDS. For variables with normal distribution (Shapiro–Wilk test), the central tendency of continuous variables was depicted using means with standard deviations such as age, ISS, GCS, and AIS. The Analysis of Variance (ANOVA) technique Bonferroni correction was used to check the means of these variables in different groups such as mechanisms of injury, age groups and ISS categories. Nonparametric statistics such as the Mann–Whitney *U* test was used for comparing medians of non-normal outcomes including ICU LOS and HLOS. A statistically significant *p*-value of 0.05 (two-sided) was used. Predictors of severe injury (ISS > 15) were performed using multivariable logistic regression analysis and data were expressed as odds ratio (OR) and 95% confidence interval (CI). Data analysis was carried out using the Statistical Package for Social Sciences version 21 (SPSS Inc., Chicago, IL).

Ethical approval for this study was granted with a waiver of consent from the Research Ethics Committee, Medical Research Center, Hamad Medical Corporation, Doha, Qatar (MRC # 01-21-230). Data were anonymously and retrospectively collected with no direct contact with the patients. The study complies with the Declaration of Helsinki and follows Strengthening the reporting of observational studies in epidemiology (STROBE) for observational cohort studies.

## Results

The study included 3,058 pediatric patients in the final analysis. Most of the patients were males (78.2%). The mean age of the study cohort was 9.3 years. The incidence rate in 2020 was estimated as 86 cases per 100,000 pediatric population in Qatar (new cases in 2020 = 453; mid-year pediatric population in 2020 = 525,584). Slightly less than half (48.9%) of the patients were nationals. Blunt injuries accounted for almost 97% of the injuries. The predominant mechanism of injury was traffic-related (31.3%) followed by fall from height (29.4%). Child abuse was documented in 0.2%. Child abuse field was added to the registry from June 2014 (based on documentation of suspected abuse and the child referred to social worker or child & women protection services). Injuries associated with fall of heavy objects were rare (2.6%). Most of the incidents occurred on the roads or streets (52.9%) followed by in-home injuries (25.8%). Injuries related to recreational activities or sports represented 15.6%. From the ED, less than 10% of the patients were transferred to the operating room, and 17.5% were admitted to the Intensive Care Unit (TICU). On the other hand, more than half (51.8%) of the patients were transferred to the surgical ward, and 9.8% were discharged home from the ED (Table 1).

**Table 1:** Demographics and characteristics of pediatric traumatic injuries, disposition, and clinical outcome (Jan 2012–Jan 2021).

Variables	Value	Variables	Value
**Male gender**	2,392 (78.2%)	**Head AIS (mean ** **±** ** SD)**	3.0 ± 1.1
**Age (years; mean ** **±** ** SD)**	9.3 ± 5.7	**Chest AIS (mean ** **±** ** SD)**	2.7 ± 0.8
**Blunt Injury**	2,949 (96.5%)	**Abdominal AIS (mean ** **±** ** SD)**	2.6 ± 0.9
**Penetrating Injury**	49 (1.6%)	**Pelvic AIS (mean ** **±** ** SD)**	2.2 ± 0.5
**Mechanism of injury**		**Upper extremity AIS (mean ** **±** ** SD)**	2.0 ± 0.3
Traffic related accidents	956 (31.3%)	**Lower extremity AIS (mean ** **±** ** SD)**	2.6 ± 0.6
Pedestrian	384 (12.6%)	**Injured body region**	
Fall from height	900 (29.4%)	Head	1,187 (38.8%)
Fall of heavy object	81 (2.6%)	Chest	610 (19.9%)
Others	737 (24.1%)	Abdomen	399 (13.0%)
**Location of injury**		Pelvis	314 (10.3%)
Home	714 (25.8%)	Upper extremity injury	481 (15.7%)
Recreational/sports	430 (15.6%)	Lower extremity injury	570 (18.6%)
Street	1,463 (52.9%)	**Intubation**	440 (14.4%)
Other	156 (5.6%)	**ORIF**	270 (8.8%)
**Mode Of transport**		**Close reduction**	249 (8.1%)
Ground ambulance	2,373 (77.9%)	**Internal Fixation**	126 (4.1%)
Private vehicle	372 (12.2%)	**Debridement**	187 (6.1%)
Air ambulance	302 (9.9%)	**Craniotomy/Craniectomy**	67 (2.2%)
**ED Disposition**		**Exploratory laparotomy**	58 (1.9%)
Operating room	284 (9.3%)	**Chest Tube**	164 (5.4%)
Trauma ICU	533 (17.5%)	**Blood transfusion**	355 (11.6%)
Floor	1,578 (51.8%)	**MTP activation**	52 (1.7%)
Died	70 (2.3%)	**In-hospital Complications**	
Home	299 (9.8%)	VAP	64 (2.1%)
Observation	191 (6.3%)	Sepsis	15 (0.5%)
HDU	56 (1.8%)	ARDS	13 (0.4%)
Transfer	34 (1.1%)	**Ventilator days**	1 (IQR, 0–2)
**Systolic blood pressure at ED**	114 ± 25.8	**ICU LOS**	0 (IQR, 0–1)
**Diastolic blood pressure at ED**	68.7 ± 17.6	**HLOS**	3 (IQR, 1–7)
**Respiratory rate at ED**	21.8 ± 7.1	**In-hospital mortality**	116 (3.8%)
**ISS (median, IQR)**	9 (4–14)	**Child abuse** ^a^	6/2,367 (0.19%)
**GCS at scene (median, IQR)**	15 (15–15)		
**GCS at emergency department (median, IQR)**	15 (15–15)		

SD, standard deviation; ICU, intensive care unit; HDU, high dependent unit; ED, emergency department; Valid percentage used; ISS, injury severity score; GCS, glasgow coma score; AIS, abbreviated injury score; ORIF, open reduction internal fixation; MTP, massive transfusion protocol; ARDS, acute respiratory distress syndrome; ICU, intensive care unit; HLOS, hospital length of stay; valid percentage used.

^a^ Child abuse field was added to the registry from June 2014 (based on documentation of suspected abuse and the child referred to social worker or child & women protection services).

The median ISS (interquartile range; IQR) was 9 (4–14) and GCS was 15 (IQR, 15–15) with the most severe injuries being those to the head with an AIS score of 3. There were 2,555 patients who had mild GCS (85.7%), 125 had moderate GCS (4.2%) and 302 had severe GCS (10.1%). Nearly 39% of the patients experienced head injuries followed by chest injuries (20%). Almost 15% of the patients were intubated. Blood transfusion was required in almost 12% of the patients and MTP was activated in less than 2 percent. In-hospital complications were rare; 2% had VAP whereas sepsis and ARDS occurred in less than 1% of cases. The median HLOS was 3 days. Of the total number of patients, 116 (3.8%) died in the hospital (Table 1).

Table 2 shows patients and injury characteristics and outcomes based on the age groups within pediatric age range. Males with age ranges from 15 to 18 years old were more likely to be injured. There was a significant difference in age by the mechanism of injuries: the victims of traffic-related accidents were older (mostly the 15–18 years old age group), whereas fall of objects was the most frequent mechanism among the 0–4 years age group (*p* = 0.001). In addition, the 0–4 years age group more frequently had fall injuries than any other age-group (*p* = 0.001). In traffic-related injuries, the ISS, and head AIS were significantly more frequent when compared to other mechanisms of injury (*p* = 0.001). On the other hand, head injuries were more frequently associated with falls (53%). Chest and abdominal injuries were more common in patients with traffic-related injuries; 33% and 17% respectively (Table 3).

**Table 2:** Characteristics of pediatric trauma patients and outcomes based on the age groups (*N* = 3,058).

Variable	0–4 years (*n* = 888, 29.0%)	5–9 years (*n* = 712, 23.3%)	10–14 years (*n* = 645, 21.1%)	15–18 years (*n* = 813, 26.6%)	*p*-value
Males (%)	584 (65.8)	547 (76.8)	534 (82.8)	727 (89.4)	0.001
**Mechanism of injury (%)**					
MVC	437 (49.2)	260 (36.5)	138 (21.4)	65 (8.0)	0.001
Fall from height	139 (15.7)	135 (19.0)	177 (27.4)	505 (62.1)	
Pedestrian hit	148 (16.7)	155 (21.8)	49 (7.6)	32 (3.9)	
Fall of heavy objects	45 (5.1)	25 (3.5)	7 (1.1)	4 (0.5)	
Other	119 (13.4)	137 (19.2)	274 (42.5)	207 (25.5)	
**Location of injury (%)**					
Home	399 (53.1)	195 (30.2)	70 (12.1)	50 (6.3)	0.001
Streets	280 (37.3)	324 (59.2)	274 (47.4)	585 (74.1)	
Sport related	40 (5.3)	80 (12.4)	182 (31.5)	128 (16.2)	
Other	32 (4.3)	46 (7.1)	52 (9.0)	26 (3.3)	
**Child abuse (*n* = 2,367)**	4/648 (0.62%)	0/566 (0.0%)	1/528 (0.19%)	1/625 (0.16%)	0.43
**ISS (Median & IQR)**	6 (1–10)	5 (2–10)	8.5 (4–13)	10 (5–17)	0.001
**Head injury**	427 (48.1)	281 (39.5)	223 (34.6)	256 (31.5)	0.001
**HLOS (Median & IQR)**	2 (1–4)	2 (1–4)	3 (1–8)	5 (2–13)	0.001
**Mortality**	39 (4.4)	21 (2.9)	19 (2.9)	37 (4.6)	0.19

MVC, motor vehicle crash; ISS, injury severity score; IQR, interquartile range; HLOS, hospital length of stay.

**Table 3:** Demographics and characteristics of pediatric traumatic injuries by mechanism of injury.

	Traffic related accidents (*n* = 956, 31.3%)	Pedestrian (*n* = 384, 12.6%)	Falls (*n* = 900, 29.4%)	Falling objects (*n* = 81, 2.6%)	Others (*n* = 737, 24.1%)	*p*-value
**Males**	793 (82.9%)	307 (79.9%)	636 (70.7%)	49 (60.5%)	607 (82.4%)	0.001
**Age (mean **±** SD)**	12.5 ± 5.5	6.7 ± 4.3	6.0 ± 4.6	5.3 ± 3.8	10.9 ± 5.0	0.001
**ISS (median & IQR)**	10 (5–17)	5 (1–13)	5 (2–10)	9 (4–10)	9 (4–13)	0.001
**GCS ED (median & IQR)**	15 (14–15)	15 (15–15)	15 (15–15)	15 (15–15)	15 (15–15)	0.001
**Head AIS**	3.3 ± 1.1	3.0 ± 1.3	2.7 ± 1.1	2.9 ± 0.9	3.1 ± 1.1	0.001
**Chest AIS**	2.6 ± 0.7	2.8 ± 0.9	2.5 ± 0.6	2.7 ± 1.1	2.8 ± 0.9	0.11
**Pelvic AIS**	2.2 ± 0.4	2.2 ± 0.7	2.2 ± 0.5	2 ± 0.0	2.1 ± 0.4	0.7
**Injured region (%)**						
Head	331 (34.6%)	133 (34.6%)	480 (53.3%)	32 (39.5%)	211 (28.6%)	0.001
Chest	316 (33.1%)	79 (20.6%)	69 (7.7%)	11 (13.6%)	135 (18.3%)	0.001
Abdomen	166 (17.4%)	48 (12.5%)	68 (7.6%)	8 (9.9%)	109 (14.8%)	0.001
Upper extremity injury	241 (25.2%)	39 (10.2%)	78 (8.7%)	4 (4.9%)	119 (16.1%)	0.001
Lower extremity injury	244 (25.5%)	94 (24.5%)	111 (12.3%)	18 (22.2%)	103 (14.0%)	0.001

SD, standard deviation; ISS, injury severity score; GCS, glasgow coma score; AIS, abbreviated injury score.

Table 4 shows that intubation was performed more frequently in traffic-related injuries when compared to other causes of injuries. Other interventions included ORIF, debridement, craniotomy or craniectomy, exploratory laparotomy and chest tube insertion significantly varied with mechanism of injury being more likely to be performed in patients with traffic-related injuries. Obviously, blood transfusion including MTP were more common among this group of patients (*p* = 0.001). In-hospital complications such as VAP and sepsis also significantly varied by mechanism of injury, whereas the occurrence of ARDS was comparable. Incidence of VAP and sepsis was higher among traffic-related accident victims. HLOS was significantly higher in this patient group. However, the mortality rate was higher in patients with pedestrian injuries (8.3%) (Table 4).

**Table 4:** Interventions, complications, and outcomes of pediatric traumatic injuries by mechanism of injury.

	Traffic related accidents	Pedestrian	Fall from height	Fall of heavy object	Others	P
**Interventions (%)**						
Intubation	208 (21.8%)	56 (14.6%)	67 (7.4%)	10 (12.3%)	99 (13.4%)	0.001
ORIF	155 (16.2%)	22 (5.7%)	36 (4.0%)	5 (6.2%)	52 (7.1%)	0.001
Close reduction	91 (9.5%)	28 (7.3%)	74 (8.2%)	8 (9.9%)	48 (6.5%)	0.22
Internal Fixation	77 (8.1%)	13 (3.4%)	15 (1.7%)	0	21 (2.8%)	0.001
Debridement	72 (7.5%)	24 (6.3%)	16 (1.8%)	5 (6.2%)	70 (9.5%)	0.001
Craniotomy/Craniectomy	26 (2.7%)	2 (0.5%)	27 (3.0%)	2 (2.5%)	10 (1.4%)	0.02
Exploratory laparotomy	36 (3.8%)	7 (1.8%)	1 (0.1%)	1 (1.2%)	13 (1.8%)	0.001
Chest Tube	87 (9.1%)	25 (6.5%)	13 (1.4%)	2 (2.5%)	37 (5.0%)	0.001
**Blood Transfusion (%)**	197 (20.6%)	46 (12.0%)	47 (5.2%)	9 (11.1%)	56 (7.6%)	0.001
**MTP**	36 (3.8%)	6 (1.6%)	2 (0.2%)	1 (1.2%)	7 (0.9%)	0.001
**In-hospital Complications (%)**						
VAP	44 (4.6%)	1 (0.3%)	5 (0.6%)	1 (1.2%)	13 (1.8%)	0.001
Sepsis	11 (1.2%)	0	1 (0.1%)	0	3 (0.4%)	0.009
ARDS	6 (0.6%)	1 (0.3%)	0	0	6 (0.8%)	0.09
**ICU LOS (median)**	1 (IQR, 1–2)	0 (IQR, 0–1)	1 (IQR, 1–1)	0 (IQR, 0–1)	1 (IQR, 0.65–1)	0.001
**HLOS (median)**	3 (IQR, 1–7)	1 (IQR, 1–3)	2 (IQR, 1–3)	1 (IQR, 1–1.75)	1 (IQR, 1–3)	0.001
**In-hospital mortality**	40 (4.2%)	32 (8.3%)	7 (0.8%)	5 (6.2%)	32 (4.3%)	0.001

ORIF, open reduction internal fixation; MTP, massive transfusion protocol; VAP, ventilator-associated pneumonia; ARDS, acute respiratory distress syndrome; ICU, intensive care unit; LOS, length of stay; HLOS, hospital length of stay.

Table 2 demonstrates that there was no significant difference in in-hospital mortality by age group, however, Table 4 shows that pedestrian injury victims were more likely to die in hospital when compared to other mechanisms of injuries. Notably, the case fatality rate (CFR) was higher among females (5% vs. 3.5%). In addition, the CFR was higher in the 15–18 years age-group. Also, the age-group of 4 years and below also had a similarly higher CFR (<1 year = 4.2% and 1–4 years = 4.4%).

A comparison between the five ISS groups among the pediatric population (Table 5) shows that 47% of the cohort had mild injury severity who were younger, injured at home, injured during recreational activities, injured by falls or by assault. Whereas more severe injuries were related to RTI and associated with higher mortality. Multivariable regression analysis for prediction of severe injury (ISS > 15) showed that, after gender adjustment, age 10–14 (OR: 1.45; 95% CI: 1.068–1.973), age 15–18 (OR: 2.27; 95% CI: 1.718–3.012) and RTI (OR: 2.05; 95% CI: 1.002–4.197) were independent predictors for more severe injuries (Table 6).

**Table 5:** Comparison based on the severity of injury (ISS)^a^.

	Mild (1–8)	Moderate (9–15)	Severe (16–24)	Very severe (25–49)	Critical (50–75)	*p*-value
Number (%)	1,400 (47.6%)	942 (32%)	324 (11%)	256 (8.7%)	18 (0.6%)	
Age in years	8.5 ± 5.4	9.5 ± 5.8	11.7 ± 5.7	10.7 ± 6.1	10.8 ± 6.2	0.001
Gender (Males)	23.3%	20.1%	15.7%	24.2%	16.7%	0.02
**Location of injury:**						
At home	28.6%	25.5%	16.1%	22.3%	23.5%	0.001
Street injury	48.9%	51.5%	63.3%	67.6%	70.6%	0.001
Recreational area	16%	17.6%	16.4%	7.3%	5.9%	0.001
**Mechanism of injury**:						0.001
Falls	33.6%	29.1%	17.9%	17.6%	0.0%	
RTI	40%	44.5%	58%	64.1%	66.7%	
Assault	2.4%	2.1%	1.9%	3.1%	0.0%	
Recreational	17%	15.8%	17.3%	8.6%	11.1%	
Fall of heavy object	2.5%	3.6%	1.9%	1.2%	0.0%	
**Hospital Mortality (*n* = 89)**	0.4%	1.9%	2.8%	18.7%	50%	0.001

^a^ ISS data are available for 2,940 patients.

**Table 6:** Multivariable analysis to predict severe injury (ISS > 15) in pediatric population.

Variable	*p*-value	Odds ratio	95% confidence interval
**Gender**	0.588	1.071	0.836–1.371
**Mechanism of injury:**			
Fall of heavy object	Reference		
Fall from height	0.967	1.015	0.490–2.102
Road traffic injury	0.049	2.050	1.002–4.197
Recreational injury	0.718	1.149	0.541–2.437
Assault or self-inflicted	0.550	1.327	0.525–3.493
**Age groups:**			
Age 0–4 years	Reference		
Age 5–9 years	0.855	0.972	0.716–1.319
Age 10–14 years	0.017	1.452	1.068–1.973
Age 15–18 years	0.001	2.275	1.718–3.012

## Discussion

The present study describes the epidemiology and outcomes of pediatric trauma in a level 1 trauma center in Qatar between 2012 and 2021. This is the largest study in our region in the Arab Middle East that addresses the pattern of injury among different age groups within the pediatric population. Children aged 18 and below represent almost 16% of the population in Qatar. Previously, Alyafei et al. (4) published a similar study based on the same data source, but it was only for a one-year duration (2011). The mean age of the victims was 10 years and the most common mechanism of injuries varied in different age groups. Nearly 83% of them were males and the mortality rate was 1.8% (4). The present study includes patient data over nine years and collects additional relevant variables, and therefore can be considered as a substantial update of the previous study. The current study demonstrates that approximately 250 pediatric trauma cases per year were presented to the trauma centre. Most of these pediatric cases were males aged less than 10 years. Traffic-related and fall-related injuries accounted for more than 60% of the injuries. One out of six patients required ICU admission. Nearly 40% were victims of head injuries. An ISS > 15 often defines severe injury (9, 10), 20.3% of our cohort had severe injury with a mean age of 11 ± 6 and 9.5% had ISS of ≥25. The predictors of severe injury were age (10 years old and above) and RTI. The in-hospital mortality rate among the pediatric trauma population was less than 4%. The case fatality rate was higher among females, and in the age group between 15 and 18 years, and those who are less than 4 years old.

Previous studies conducted across the Arabian Gulf region reported that male child is more likely to incur traumatic injuries, perhaps because of greater involvement in physical contact sports, risk-taking behaviour, and higher levels of aggression (5, 6, 11–19). Consistent with these findings, our results also demonstrated male predominance in pediatric trauma.

In our study, the main mechanisms of injury were MVCs and falls, which contributed almost equally in proportion, 31% and 29% respectively. Pedestrian injuries were more fatal and accounted for almost one out of eight cases. Consunji et al. (5) previously studied pediatric RTIs in Qatar and demonstrated that 54% of the RTIS were due to MVCs and a quarter of RTIs were due to pedestrian injuries. Notably, 14% of the total RTIs were caused by All-Terrain Vehicle-related accidents. Grivna et al. (12) studied pediatric trauma patients (0–19 years) injured by RTIs in the United Arab Emirates (UAE) and found that MVCs (70%) were the main cause followed by pedestrian injuries (15%). The most common mechanism of RTI in a study from the UAE was rollover of vehicle (37%) followed by front impact collision (32%). Multiple studies have shown that MVC was the most frequent cause of pediatric head trauma in the Kingdom of Saudi Arabia (KSA) (13, 19). Al-Sarheed et al. (18) reported that the most common cause of spinal injury was MVC, followed by pedestrian accidents and falls in patients aged 15 years and below.

On the other hand, Alkhamis and Abdulkader (17) reported that the majority of pediatric injuries occurred at home and accidental falls were the most frequent cause of injury followed by burns. This study was a cross-sectional survey among parents accompanying children aged ≤12 years to the pediatric outpatient clinics of a tertiary care hospital in KSA. Only two out of 283 patients had experienced MVC, while fall was reported in 63 percent. Al Ateeq et al. (16) demonstrated that the leading cause of pediatric trauma was fall followed by hot liquids and chemical exposure. This study included pediatric patients up to the age of 14 years who had non-traffic unintentional injuries and were admitted to the ED. The study from Oman reported that nearly half of the pediatric injuries (patients between 0 and 15 years old) were fall related, followed by RTIs and exposure to inanimate mechanical forces (1).

The mechanism of injury varies within the pediatric age-groups. Our study found that RTIs were more frequent in teenagers (15–18 years age) than any other age-group. Additionally, infants/toddlers (≤4 years age) were more commonly injured by falling objects. Furthermore, the prevalence of falls in this age-group was significantly higher when compared to other age-groups. A recent study based on the pediatric trauma registry data in Bahrain revealed that fall injury was most predominant in infants under the age of one year followed by children under the age of 5 years old (20). A recent study by Mekkodathil et al. (21) showed that one out of five fall-associated injuries among the general population in Qatar occurs in the pediatric population. These findings are in line with El-Menyar et al. (22) study which revealed that MVC associated traumatic brain injury (TBI) was more frequently reported among teenagers (77%) and adolescents (10–14 years; 48%) whereas fall was the commonest cause of TBI in infants/toddlers (51%) and in middle childhood (5–9 years) (30%).

The overall in-hospital mortality rate in the study cohort was 3.8%, and mortality was significantly higher after pedestrian injury (8.3%) followed by falling objects (6.2%) and RTI (4.2%). The CFR was higher among females when compared to males (5% vs. 3.5%). It was also higher in teenagers as well as infants/toddlers, however the predominant mechanisms of injuries leading to death varies by age, MOI and ISS.

Previous research focused on RTIs in Qatar reported the mortality rate was 3.4% in which the MVC-associated mortality rate represented 6.2% (5). Our study shows that this rate reduced to almost by one-third over the following years. However, there was an alarming rise in pedestrian injuries and related mortality, with reference to the Consunji et al. (5) study based on the trauma registry data from 2010 to 2012. El-Menyar et al. (22) study among the pediatric TBI patients in Qatar also revealed that pedestrian injury was associated with a higher CFR (22.2%), followed by MVC (CFR = 12.7%) and falls (CFR = 8.3%).

The UAE-based study reported that the mortality rate among pediatric vehicle occupants was 4.1% (12). The results from the household survey in the KSA revealed that the mortality rate among injured children was 1.5%. In addition, a low mortality rate (1.2%) was also reported by Al Ateeq et al. (16) in non-traffic related injuries in the KSA. In contrast, the retrospective study conducted over 10 years duration among the pediatric patients with traumatic head injury in the KSA reported that the most common cause of injury was MVC, and the overall mortality was nearly 15%.

The severity of MVC injuries could be reduced by stricter adherence to correct use of seatbelts and other safety features such as airbags and electronic stability controls. Prior studies reported a lower level of child-restraint use in the UAE, Qatar, Saudi Arabia and Bahrain (19), in addition to the evidence of greater usage of mobile phones while driving (6, 12, 13, 20, 23).

Introducing an inclusive trauma system in the Netherlands and performance improvement program in Michigan, USA, were found to reduce the mortality by 16% and 12% respectively (24, 25). A survey of western trauma hospitals found that quality improvement programs were strong predictors of survival (26). Substantial evidence currently exists supporting the relationship between adequately resourced trauma centres operating within regionalised trauma systems and improved mortality in severely injured patients. Haut et. al (27). addressed the advantages of reporting long term trends and time series analyses including the ability to demonstrate sustained improvements in patient outcomes.

Should pediatric injuries be treated in specialized pediatric Trauma centres (PTCs)? Only 10% of pediatric trauma patients are currently treated at PTCs (28). Improved outcomes at such centres can be understood to result from greater experience gained over time. When compared to children admitted to the Adult Trauma Care, those who were admitted to the PTC were significantly less likely to end up in ICU (17% vs. 23%) (29). Previous research has noted that poorer outcomes resulted when young children were treated in the adult emergency settings (30). MacKenzie et al. (31) identified a problem with the integration of trauma systems and supported the call for improvements across the region, and more recently, they suggested such issues as one of reasons that rise the caseload of pediatric TBI.

According to Consunji et al. (5), the ratio of adult to pediatric trauma cases in Qatar is around 10:1. As the national population of Qatar is only 2.8 million, this means that there is an underexposure of pediatric surgeons to cases of trauma. This can be particularly problematic in cases of severe and multiple injury. Ideally, sharing knowledge and experience between pediatric and adult trauma surgeons should be facilitated by enabling them to work side by side and gain valuable insights from each other. The trauma surgeon will benefit from advice and guidance when confronted with a pediatric patient, and may, in turn offer support when the pediatric surgeon is faced with a challenging trauma case.

The HTC is based at the Hamad General Hospital and potentially benefits from the presence of all subspecialities in proximity. The other centre may have several logistical issues preventing the service from offering the best possible care. One is triaging pediatric trauma patients; another is communication, which may cause problems in locating the appropriate lead physician. A third obstacle is the physical distance between departments which may delay prompt care services. The practice of both pediatric and adult trauma surgeons would be enhanced by sharing knowledge and experience. Such an approach would be beneficial in improving the treatment and outcomes for pediatric trauma patients.

### Recommendations

The study findings serve as an important base line for future studies. There is a need for targeted practical strategies that benefit the country as well as neighboring regions experiencing similar issues. Initially, children aged between 10 and 18 years old may be targeted for a particular prevention and intervention strategies with increasing the awareness for both the public and professionals. Firstly, through education campaigns on safety awareness and prevention measures including parents, pupils as well as school curricula. Additionally, non-governmental advisory committees may train youth of a similar age, previous crash victims, and their parents as voluntary ambassadors, in accord with the sequalae effect. Secondly, tailored simulation programs of real scenarios in hospitals and universities. Such simulations for adult surgeons increase their competence in dealing with pediatric age-groups. Thirdly, the distribution of clear management guidelines and integration of systems, especially for patients aged 12–18 who may be treated by both pediatric and adult surgeons. Finally, legislation must be strictly enforced when it comes to safety measures which require open lines of communication between healthcare personnel and government representatives. All the above should be continuously audited to ensure progress and success in decreasing trauma injuries and mortalities within set time frames.

### Limitations

As this is a single centre and retrospective analysis; biases are unavoidable, which may affect the generalizability of the findings. Our study does not assess the long-term functional outcomes as prior studies (32) have reported an increased risk of psychosocial and cognitive problems among children with minor head injuries. Child abuse is under-reported in this database.

## Conclusion

Almost one-fifth of the trauma admissions at the level 1 trauma centre in Qatar are among the pediatric population. Developing a policy guided by understanding the age and mechanism-specific patterns of traumatic injuries remain crucial.

## Data Availability

All data generated or analyzed during this study are included in this article results and tables. Data are accessible upon permission and agreement with the Medical Research Centre, Qatar (mrchelp@hamad.qa).
